# The Genetics of Primary Biliary Cholangitis: A GWAS and Post-GWAS Update

**DOI:** 10.3390/genes14020405

**Published:** 2023-02-03

**Authors:** Yuki Hitomi, Minoru Nakamura

**Affiliations:** 1 Department of Human Genetics, Research Institute, National Center for Global Health and Medicine, 1-21-1 Toyama, Shinjuku-ku, Tokyo 162-8655, Japan; 2 Clinical Research Center, National Hospital Organization (NHO) Nagasaki Medical Center, 2-1001-1 Kubara, Omura 856-8562, Japan; 3 Department of Hepatology, Nagasaki University Graduate School of Biomedical Sciences, 2-1001-1 Kubara, Omura 856-8562, Japan; 4 Headquarters of PBC Research in NHO Study Group for Liver Disease in Japan (NHOSLJ), Clinical Research Center, National Hospital Organization (NHO) Nagasaki Medical Center, 2-1001-1 Kubara, Omura 856-8562, Japan

**Keywords:** primary biliary cholangitis (PBC), organ-specific autoimmune disease, polygenic complex trait, genetic factor, genome-wide association study (GWAS), disease susceptibility gene, post-GWAS study, disease pathogenesis

## Abstract

Primary biliary cholangitis (PBC) is a chronic, progressive cholestatic liver disease in which the small intrahepatic bile ducts are destroyed by autoimmune reactions. Among autoimmune diseases, which are polygenic complex traits caused by the combined contribution of genetic and environmental factors, PBC exhibits the strongest involvement of genetic heritability in disease development. As at December 2022, genome-wide association studies (GWASs) and associated meta-analyses identified approximately 70 PBC susceptibility gene loci in various populations, including those of European and East Asian descent. However, the molecular mechanisms through which these susceptibility loci affect the pathogenesis of PBC are not fully understood. This study provides an overview of current data regarding the genetic factors of PBC as well as post-GWAS approaches to identifying primary functional variants and effector genes in disease-susceptibility loci. Possible mechanisms of these genetic factors in the development of PBC are also discussed, focusing on four major disease pathways identified by in silico gene set analyses, namely, (1) antigen presentation by human leukocyte antigens, (2) interleukin-12-related pathways, (3) cellular responses to tumor necrosis factor, and (4) B cell activation, maturation, and differentiation pathways.

## 1. Introduction

Primary biliary cholangitis (PBC) is a chronic cholestatic liver disease characterized by the destruction of the intrahepatic bile ducts, portal hypertension, the development of biliary cirrhosis, and ultimately, hepatic failure. Histologically, PBC is characterized by chronic, non-suppurative destructive cholangitis, ductopenia, interface hepatitis, and fibrosis [1,2,3,4]. Although recent studies have reported a good prognosis for the majority of PBC patients treated with ursodeoxycholic acid, obeticholic acid, and bezafibrate, approximately 10% to 20% of patients do not respond to these agents, eventually progressing to end-stage hepatic failure [5]. Therefore, novel therapies are needed for these treatment-resistant cases.

The destruction of bile ducts is mediated by autoimmune responses, including both adaptive immune responses (including CD4^+^ T cells, CD8^+^ T cells, and B cells) and innate immune responses (including natural killer [NK] cells), against biliary epithelial cells (BECs) [4,6,7]. Therefore, PBC is considered an organ-specific autoimmune disease. PBC also has the following features in common with autoimmune diseases. First, PBC exhibits a striking female predominance as it frequently occurs in middle-aged and older women, similarly to other autoimmune diseases [8]. Second, anti-mitochondrial antibodies (AMAs) and anti-nuclear antibodies, such as anti-gp210 antibodies, anti-centromere antibodies, and anti-sp100 antibodies, are specifically detected in PBC patients [9]. The E2 domain of the pyruvate dehydrogenase complex (PDC-E2) was reported to be the primary autoantigen to which more than 90% of antibodies in PBC patients’ serum react [10]. Infiltrations of autoreactive CD4^+^ and CD8^+^ T cells that react with PDC-E2 also contribute to lymphocytic inflammation around portal tracts and BECs [11]. Third, PBC shows a high rate of complications with other autoimmune diseases. In particular, Sjogren’s syndrome, which is characterized by autoimmune-mediated inflammation of the salivary glands, has been frequently reported to co-exist with PBC [12].

PBC is a polygenic complex trait that develops due to the combined contribution of genetic and environmental factors. The strong involvement of heritability in the development of PBC has been highlighted by previous family-based epidemiologic studies. Indeed, the higher estimated sibling–relative risk (with a lambda value of 10.5) and the higher concordance rate (0.63) in monozygotic twins compared with dizygotic twins, indicate the involvement of genetic factors in the development of PBC [13,14]. These values are the highest among autoimmune diseases [15].

Beginning in the early 2000s, the genome-wide association study (GWAS) has become the primary comprehensive approach for investigating disease-susceptibility genes for polygenic complex traits. The GWAS is a statistical genetics method that identifies disease-susceptibility gene loci by conducting case-control association studies using commercially available DNA arrays that can simultaneously analyze 500,000 to 5 million variants in samples from affected patients and controls. The association of these variants is reflected by several “primary functional variants” that exhibit linkage disequilibrium (LD) with them. As at December 2022, a total of 6165 studies and 452,329 unique variant-trait associations was registered in the National Human Genome Research Institute Catalog of published GWASs (http://www.ebi.ac.uk/gwas/, accessed on 1 December 2022) [16]. Among the polygenic traits in this database, a total of 13 studies and 264 unique variant-trait associations linked to susceptibility to PBC have been registered. In particular, *Human Leukocyte Antigen (HLA)* loci are strongly associated with susceptibility to PBC as well as various other autoimmune or immune-related diseases [17]. However, the molecular mechanisms through which these susceptibility loci affect the pathogenesis of PBC are not fully understood.

This study provides an overview of the latest reported genetic factors associated with PBC, including the *HLA* locus and outside *HLA* loci, from both a PBC-specific and non-PBC-specific perspective. We also discuss the possible contribution of these genetic factors to the pathogenesis of PBC based on post-GWAS approaches using in silico gene set analysis, the integration of GWAS and transcriptome data, and in silico drug efficacy screening. Furthermore, we identify and evaluate functional variants in disease-susceptibility loci.

## 2. Genetic Factors Associated with PBC

### 2.1. Human Leukocyte Antigen (HLA)

Class II HLA are signal transduction molecules that present specific antigenic peptides to T cell antigen receptors expressed on CD4^+^ helper T cells. The *HLA* class II locus on chromosome 6 includes groups of genes encoding HLA class II proteins that are strongly associated with susceptibility to various autoimmune or immune-related diseases [17]. As *HLA* loci are highly polymorphic due to natural selection driven by a wide range of pathogens [18], accurate detection of disease-susceptibility loci can be challenging. For example, deviations from the Hardy–Weinberg equilibrium in case-control association studies for each single-nucleotide polymorphism (SNP) in the *HLA-DRB* locus are caused by gene copy number variations. Therefore, to identify disease-susceptibility alleles in *HLA* loci, it is necessary to perform *HLA* allele typing using approaches such as the Luminex sequence-specific oligo method or the next-generation sequencing-based *HLA* allele typing method.

In the pre-GWAS era, many studies were conducted examining the association between susceptibility to PBC and *HLA* class II alleles. In European populations, including the US, UK, Italy, and Sweden, HLA-DQA1*04:01, HLA-DQB1*04:02, and HLA-DRB1*08:01 were reported as predisposing alleles (odds ratio [OR]: 2.90–8.16), whereas HLA-DQB1*03:01 was identified as a protective allele (OR: 0.47–0.70) [19,20,21,22,23]. In Asian populations, including Japan and China, HLA-DRB1*08:03 was reported as a predisposing allele (OR: 1.77–3.17), whereas HLA-DQB1*03:01, HLA-DQB1*06:04, and HLA-DRB1*13:02 were identified as protective alleles (OR: 0.19–0.54) [24,25,26,27,28]. Multinacci et al. summarized how HLA modifications are associated with the pathogenesis of PBC [29].

Recently, tools have been established that can predict *HLA* alleles from existing GWAS data [30,31]. Furthermore, next-generation sequencer *HLA* typing methods have also been developed [30]. These approaches will help elucidate the contribution of *HLA* genes to PBC on a larger scale.

### 2.2. PBC Susceptibility Loci Outside HLA

Previous GWASs involving ImmunoChip analyses and meta-analyses in populations of European descent identified 31 PBC susceptibility loci [32,33,34,35,36,37,38,39]. The nearest genes from the lead SNPs in each locus were as follows: *MMEL1*, *IL12RB2*, *CD58*, *DENND1B*, *STAT4*, *PLCL2*, *TIMMDC1*, *IL12A*, *NFKB1*, *IL7R*, *IL12B*, *HLA*, *LINC03004*, *ELMO1*, *TNPO3*, *CCDC88B*, *CXCR5*, *TNFRSF1A*, *ATXN2*, *TNFSF11*, *DLEU1*, *RAD51B*, *EXOC3L4*, *CLEC16A*, *IL21R*, *IRF8*, *IKZF3*, *HROB*, *TYK2*, *SPIB*, and *SYNGR1* (Table 1). Additionally, other GWASs identified 14 (nearest genes: *CD58*, *STAT4*, *CD28*, *TIMMDC1*, *IL12A*, *NFKB1*, *IL7R*, *HLA*, *TNFSF15*, *POU2AF1*, *CXCR5*, *IL21R*, *IKZF3*, and *SYNGR1*) PBC susceptibility loci in East Asian populations (Table 1) [40,41,42,43]. Recently, the largest genome-wide meta-analysis (meta-GWAS) combining new and previously reported genotype data for 10,516 cases and 20,772 controls from five European and two East Asian cohorts was conducted by our international PBC research group. This study identified 22 novel PBC susceptibility gene loci (nearest genes: *FCLR3*, *CACNA1S*, *LINC00299*, *DNMT3A*, *TMEM163*, *RARB*, *TET2*, *ST8SIA4*, *NDFIP1*, *ATG5*, *CCR6*, *ITGB8*, *ZC3HAV1*, *MYC*, *ANP32B*, *WDFY4*, *DEAF1*, *ETS1*, *SRP54*, *RIN3*, *DPEP2*, and *CD226* on autosomes, and *GRIPAP1* on the X chromosome) (Table 1) [44,45]. Among these PBC-susceptibility loci, approximately 60% were identified only in European populations, probably due to the strong statistical power afforded by the large sample size of the European population. Conversely, *CD28*, *CCR6*, and *TNFSF15* were identified only in East Asian populations [44]. These results indicate that population-specific and non-specific genetic factors play a role in the development of PBC; however, the disease pathways are very similar in European and East Asian populations. 

Generally, a GWAS aims to comprehensively search for genetic factors that contribute to the onset of a disease. However, by using the same methodology, it is also possible to comprehensively explore genetic factors associated with disease severity, response to treatment, and side effects. The *IL28B* and *ITPA* genes are known to be strongly associated with response to treatment and side effects of pegylated interferon-α and ribavirin therapy for chronic hepatitis C [46,47,48,49]. Recently, genetic loci near *L2TFL1*, *FOXP4*, *TMEM65*, *OAS1*, *KANSL1*, *DPP9*, *RAVER1*, and *IFNAR2* were reported as susceptibility loci for severity to coronavirus disease 2019 (COVID-19), which is caused by severe acute respiratory syndrome coronavirus 2 (SARS-CoV-2) [50]. As for PBC, *CTSZ/NELFCD*, which has not been reported as a PBC-susceptibility locus, was associated with the progression of PBC by comparing an advanced jaundice/liver failure group with a non-advanced, early-stage group of Japanese PBC patients [51].

GWAS data can also be used to examine the effects of multiple variants that are located within a chromosomal unit region but contribute only marginally to pathogenesis. For example, *STAT4*, *ULK4*, and *KCNH5* were identified as novel Japanese PBC susceptibility loci in a study utilizing regional gene mapping, a method used to detect polygenic effects [52] (Table 1).

### 2.3. Specific and Non-Specific PBC Susceptibility Genes

Over 80% of identified PBC susceptibility gene loci overlap with loci associated with other autoimmune diseases, such as Crohn’s disease, ulcerative colitis, multiple sclerosis, systemic lupus erythematosus, autoimmune thyroid disease, rheumatoid arthritis, psoriasis, ankylosing spondylitis, systemic sclerosis, celiac disease, type 1 diabetes, Sjogren’s syndrome, vitiligo, and Bechet’s disease. Conversely, PBC susceptibility loci near *TMEM163*, *TET2*, *ITGB8*, *ZC3HAV1L*, *RIN3*, *EXOC3L4*, *DPEP2*, and *SPIB* are specific for PBC and not associated with other autoimmune diseases [16,53]. Under the genetic background in which loci are shared among various autoimmune diseases, it is assumed that the development of PBC is caused by PBC-specific genetic and environmental factors. Further cross-disease analyses are warranted to confirm this hypothesis.

## 3. Post-GWAS Studies

### 3.1. Identification of Primary Functional (Causal) Variants and Effector Genes

Within a given disease-susceptibility gene locus, the variant exhibiting the strongest association with disease susceptibility is known as the “GWAS lead variant.” The GWAS lead variant in each disease susceptibility locus often does not contribute *per se* to disease susceptibility. The lowest *p*-value for the association with disease susceptibility is frequently affected by LD with a “primary functional variant” that directly contributes to disease development. In other words, the GWAS is a means of identifying the “mapped gene,” which is the gene nearest to the GWAS lead variant. Therefore, it is necessary to identify the primary functional variant itself from among many variants in the disease-susceptibility gene locus.

**Figure 1:**
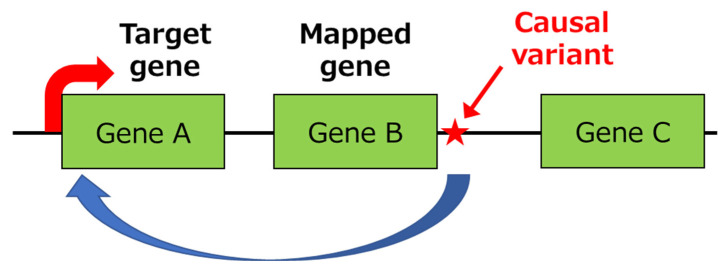
Example of the variant that regulates the expression of genes, separated from the primary functional variant by several hundred kilobases. As DNA adopts a higher-dimensional structure during transcription that associates with physically distant gene regions, primary functional variants often control the expression of other genes that are separated by several hundred kilobases (i.e., Gene A) from the mapped gene (i.e., Gene B).

Most of the primary functional variants are located in untranslated regions (UTRs) or expression regulatory regions, such as gene expression promoters and enhancers located in the intronic or intergenic region, and they do not alter the amino acid sequence of the relevant gene product. Primary functional variants usually control the expression of the mapped gene in which they are located. However, as DNA adopts a higher-dimension structure during transcription that associates with physically distant gene regions, primary functional variants often control the expression of other genes that can be more than several hundred kilobases apart (Figure 1). In this way, the gene regulated by the primary functional variant (i.e., the effector gene) sometimes does not match the mapped gene (genes shown in red in Table 1), as determined by analyses of public expression quantitative trait loci (e-QTL) databases, including GTEx [54] and ImmuNexUT [55]. Therefore, inferring an association with pathogenesis only from the mapped gene may lead to misinterpretation of disease pathways.

To date, we have identified 11 primary functional variants in 60 PBC susceptibility gene loci (Table 1). In order to identify primary functional variants from many variants that exhibit higher LD with GWAS lead SNPs, we selected candidate variants by in silico analysis. Gene expression-regulating variants are located within DNA sequences that show higher scores for both histone marks (H3K27Ac, H3K4Me1, and H3K4Me3) and histone loosening (DNase high-sensitivity sites and peak of ATAC-seq) in promoters, enhancers, and insulators. Furthermore, differences between major and minor alleles in transcription factor binding to the variant, in addition to significant e-QTL, are also characteristics of primary functional variants. Variants that regulate mRNA structure and translation are located in the 3′- and 5′-UTRs, respectively. Splicing–regulating variants are located within the splice site, exonic splicing enhancers, exonic splicing silencers, intronic splicing enhancers, or intronic splicing silencers. Candidate variants can be efficiently narrowed down by thoroughly analyzing each variant. Moreover, for the definitive identification of primary functional variants, in vitro functional evaluation of candidates selected via in silico analysis should be performed.

Using the allele knock-in version of a cell line constructed using genome-editing techniques such as CRISPR/Cas9, the endogenous effect of a primary functional variant can be evaluated without interference from other variants that show high LD. These analyses can also be used to elucidate the mechanism of pathogenesis. Among primary functional variants in each PBC susceptibility locus, approximately 40% were found to be associated with the expression of each mapped gene (Table 1, genes shown in black among “e-QTL genes”). For example, rs4979462, rs17032850, rs227361, rs9459874, and rs1012656, located in *TNFSF15*, *NFKB1*, *MANBA*, *FGFR1OP*, and *CCR6*, respectively, were identified as primary functional variants that regulate the expression of each mapped gene [56,57,58]. Alternatively, approximately 40% of primary functional variants were associated with the expression of genes other than the mapped genes (Table 1, genes shown in red among “e-QTL genes”). For example, rs12946510, rs2293370, and rs1944919, which are located near *IKZF3*, *TIMMDC1*, and *POU2AF1*, respectively, regulate the expression of *ORMDL3*, *POGLUT1*, and *COLCA1/COLCA2*, respectively [42,59,60] (Table 1). Therefore, such discrepancies between mapped genes and e-QTL genes can be corroborated by in vitro functional evaluation using genome-editing techniques. This type of functional evaluation was applied to the variants that regulate splicing. *CD28*, which was reported as a susceptibility locus for lymphocyte and eosinophil counts, multiple sclerosis, ulcerative colitis, celiac disease, rheumatoid arthritis, asthma, and PBC, has a shared disease-related primary functional variant (i.e., rs2013278) that regulates the CD28 alternative splicing that generates loss-of-function isoforms (CD28i and CD28∆ex2) [61].

### 3.2. In Silico Gene Set Analysis

Compared with monogenic diseases that are caused by mutations in a single gene within a patient’s DNA sequence, each GWAS lead variant in the disease-susceptibility gene locus will show a weak association with the risk of a polygenic complex disease. Although the *HLA* class II locus showed a relatively high OR, similarly to other autoimmune diseases, all other PBC susceptibility gene loci outside *HLA* showed an OR of less than 2.0. However, by analyzing disease-susceptibility gene loci in detail by gene set analysis, it is possible to obtain a more comprehensive understanding of disease pathways. In such cases, gene set analysis using only mapped genes may be insufficient, because primary functional variants can sometimes be associated with the expression of genes other than the mapped genes. Gerussi et al. summarized the PBC susceptibility genes as of 2021 and classified the contribution of candidate causal genes, as well as mapped genes, into interleukin (IL)-12-mediated signaling, the cellular response to tumor necrosis factor (TNF), and the activation, maturation, and differentiation of B cells [53]. 

In addition, functional enrichment analysis based on protein–protein interactions using STRING [62] was found to be very useful in further identifying disease pathways in detail. The following functional enrichment analysis using STRING on these three pathways highlighted the importance of using not only mapped genes, but also e-QTL genes.

#### 3.2.1. IL-12-Mediated Signaling Pathway

**Figure 2:**
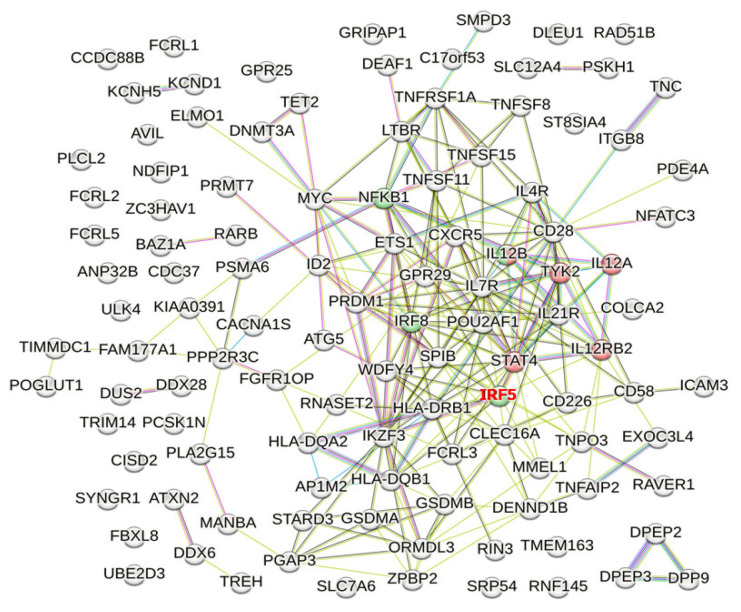
PBC susceptibility mapped genes and e-QTL genes whose protein products are involved in Gene Ontology (GO) pathways related to “IL-12-mediated signaling.” Among 59 PBC susceptibility mapped genes and 60 e-QTL genes, those defined in the GO database as “Interleukin-12-mediated signaling pathway (GO: 0035722)” and “Regulation of interleukin-12 production (GO: 0032655)” are shown as red and green nodes, respectively. However, after removal of “the e-QTL gene but not the mapped gene” (gene name shown in red), “Regulation of interleukin-12 production (GO: 0032655)” in the Gene Ontology database did not reach a level of statistical significance when using PBC susceptibility genes alone.

Interleukin-12 induces interferon (IFN)-γ production. By the enrichment analysis using STRING, the “Interleukin-12-mediated signaling pathway (GO: 0035722)” in the Gene Ontology database includes mapped PBC susceptibility genes and e-QTL genes such as *IL12A*, *IL12B*, *IL12RB2*, *STAT4*, and *TYK2* (shown as red nodes in Figure 2; *p* = 0.0034) [62]. In addition, “Regulation of interleukin-12 production (GO: 0032655)” includes genes such as *IL12B*, *NFKB1*, *IRF5*, and *IRF8* (shown as green nodes in Figure 2; *p* = 0.0472) [62]. However, *IRF5* was not a PBC susceptibility mapped gene, but an e-QTL gene. Therefore, “Regulation of interleukin-12 production (GO: 0032655)” in the Gene Ontology database did not reach a level of statistical significance when using PBC susceptibility genes alone.

Death domain receptor 3 (DR3, also known as TNFRSF25), the receptor for TNF-like cytokine 1A (TL1A; encoded by the Asian-specific PBC susceptibility gene *TNFSF15*), promotes IFN-γ production through activated CD4^+^ T cells. This suggests that TL1A is positioned in the pathway, leading to IL-12 receptor (IL12R) stimulation and IFN-γ production [63]. Additionally, studies have reported the increased expression of IFN-γ-related genes in the liver of PBC patients [64], increased expression of IFN-α/IFN-β and IFN-γ in lesions of PBC liver [65], and the appearance of PBC-like liver lesions in female mice with chronic expression of IFN-γ [66]. These data also suggest the importance of the IL-12 signaling pathway.

#### 3.2.2. Cellular Response to TNF

**Figure 3:**
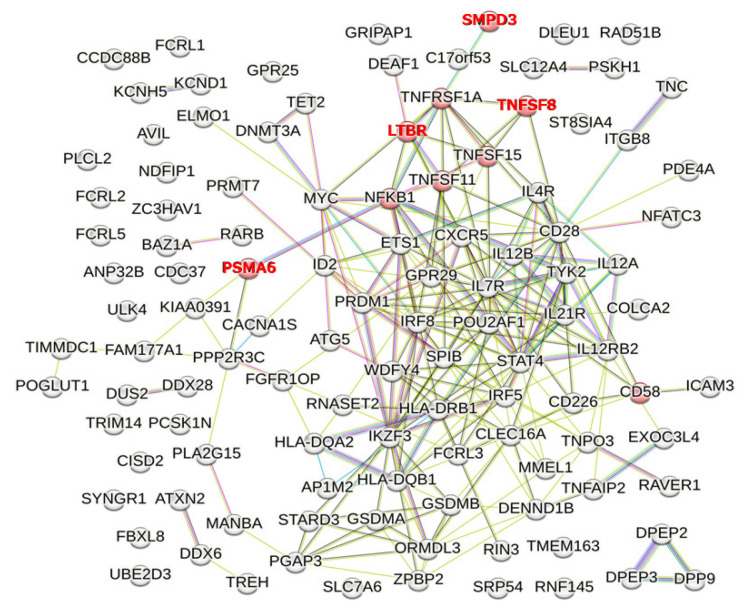
PBC susceptibility mapped genes and e-QTL genes whose protein products are involved in GO pathways related to “Cellular response to tumor necrosis factor.” Genes identified in the GO database as part of pathways associated with “Cellular response to tumor necrosis factor (GO: 0071356)” are shown as red nodes. However, after removal of “the e-QTL genes but not the mapped genes” (gene names shown in red), “Cellular response to tumor necrosis factor (GO: 0071356)” in the Gene Ontology database did not reach a level of statistical significance when using PBC susceptibility genes alone.

TNF-α itself plays a role in disrupting the barrier function of tight junctions in cholangiocytes without inducing major structural changes [67]. Additionally, each member of the TNF superfamily and TNF receptor (TNFR) superfamily plays a distinct role. By conducting enrichment analysis using STRING, the “Cellular response to tumor necrosis factor (GO: 0071356)” in the Gene Ontology database includes genes such as *TNFSF8*, *TNFSF15*, *TNFSF11*, *TNFRSF1A*, *LTBR*, *NFKB1*, *PSMA6*, *SMPD3*, and *CD58* (shown as red nodes in Figure 3; *p* = 0.0041) [62]. However, *TNFSF8, LTBR, PSMA6*, and *SMPD3* were not PBC susceptibility mapped genes, but e-QTL genes. Therefore, “Cellular response to tumor necrosis factor (GO: 0071356)” in the Gene Ontology database did not reach a level of statistical significance when using PBC susceptibility genes alone.

TNFR superfamily member 1A (TNFRSF1A, also known as TNFR1) is a receptor for TNF-α. Activated TNFRSF1A initiates caspase-8-mediated apoptosis via receptor-interacting serine/threonine–protein kinase 3 (RIPK3) [68]. TNF superfamily member 8 (TNFSF8, also known as CD30L and CD153) inhibits class-switch DNA recombination and antibody production in human IgD^+^ IgM^+^ B cells by engaging with the ligand TNFR superfamily member 8 (TNFRSF8, also known as CD30) [69]. Although TNF superfamily member 11 (TNFSF11, also known as RANKL and CD254) plays a key role in the differentiation and activation of osteoclasts, the pathway including TNFSF11 in dendritic cells (DCs) promotes naïve T cell proliferation and DC survival [70]. This pathway also regulates lymph node organogenesis and lymphocyte differentiation [71]. The lymphotoxin β receptor (also known as TNFRSF3) is involved in the thymic homing of lymphoid progenitor and peripheral antigen-presenting cells, thymocyte trafficking and shedding, differentiation of medullary thymic epithelial cells, and the establishment of central tolerance [72]. TNF-α and related molecules play important roles in several immunologic reactions related to the pathogenesis of PBC.

#### 3.2.3. Activation, Maturation, and Differentiation of B Cells

**Figure 4:**
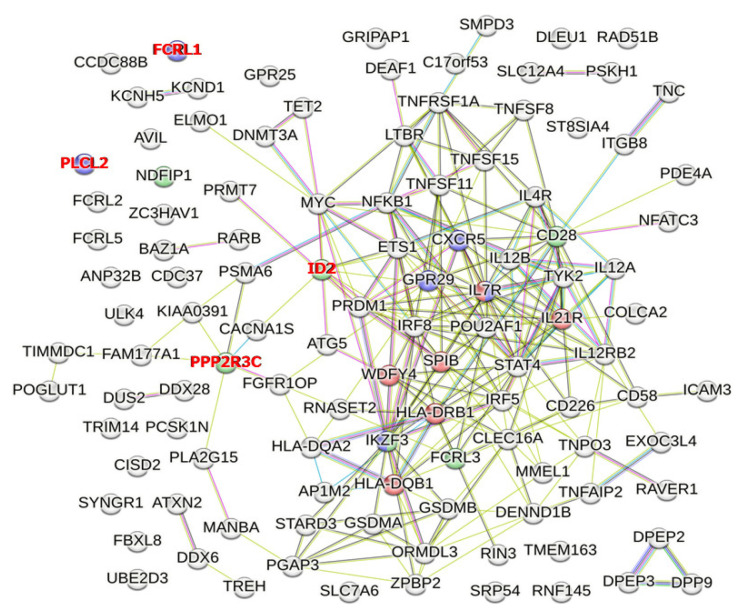
PBC susceptibility mapped genes and e-QTL genes whose protein products are involved in GO pathways related to “Activation, maturation, and differentiation of B cells.” Genes identified in the GO database as part of pathways associated with “Regulation of B cell activation (GO: 0050864),” “B cell activation (GO: 0042113),” and “B-lymphocyte cell line (BTO: 0001522)” are shown as blue, green, and red nodes, respectively. However, after removal of “the e-QTL genes but not the mapped genes” (gene names shown in red), “Regulation of B cell activation (GO: 0050864)” and “B cell activation (GO: 0042113)” in the Gene Ontology database did not reach a level of statistical significance when using PBC susceptibility genes alone.

By the enrichment analysis using STRING, the “Regulation of B cell activation (GO: 0050864)” in the Gene Ontology database includes genes such as *CD28*, *FCRL3*, *IKZF3*, *ID2*, *PPP2R3C*, and *NDFIP1* (shown as blue nodes in Figure 4; *p* = 0.0156). The “B cell activation (GO: 0042113)” in the Gene Ontology database includes genes such as *IL7R*, *CXCR5*, *CCR6*, *IKZF3*, *FCRL1*, and *PLCL2* (shown as green nodes in Figure 4; *p* = 0.0383). Finally, the “B-lymphocyte cell line (BTO: 0001522)” in the Gene Ontology database includes genes such as *HLA-DRB1*, *HLA-DQB1*, *WDFY4*, *SPIB*, *IL7R*, and *IL21R* (shown as red nodes in Figure 4; *p* = 0.0139) [62]. However, *ID2*, *PPP2R3C*, *FCRL1*, and *PLCL2* were not PBC susceptibility mapped genes, but e-QTL genes. Therefore, “Regulation of B cell activation (GO: 0050864)” and “B cell activation (GO: 0042113)” in the Gene Ontology database did not reach a level of statistical significance when using PBC susceptibility genes alone.

POU class 2 homeobox associating factor 1 (POU2AF1) and its cofactor, Spi-B transcription factor (SPIB), are essential molecules in the differentiation and maturation of B cells into plasma cells [73]. IKAROS family zinc finger 3 (IKZF3, also known as aiolos) is a transcription factor essential for B cell differentiation into plasma cells and long-term B cell memory [74]. The C-X-C motif chemokine receptor 5 (CXCR5) is involved in the homing of follicular helper T cells (i.e., it is involved in affinity selection after T cell–B cell interactions) to germinal centers, and IL-21 is a growth factor for CXCR5-positive T cells [75,76]. Additionally, certain subgroups of helper T cells, such as CCR9^+^ CXCR5^+^ cells, express more IL-7 receptor (IL7R)-α and secrete a higher proportion of IL-21, thereby inducing greater IgG production than CCR9^−^ CXCR5^−^ helper T cells [75]. Fc receptor-like (FCRL) family members are preferentially expressed on B cells and exert immunomodulatory functions involving the immunoreceptor tyrosine-based inhibitory motif (ITIM) and immunoreceptor tyrosine-based activation motif. FCRL1 has two ITIMs in its cytoplasmic tail and enhances B cell antigen receptor (BCR) signaling. In contrast, other FCRL members, including FCRL3, have an ITIM in their cytoplasmic tail and inhibit BCR signaling [77,78]. These results strongly suggest that B cell activation, maturation, and differentiation pathways are actively involved in the development of PBC.

### 3.3. Integration of GWAS and Transcriptome Data

Based on gene ontology analyses, PBC susceptibility mapped genes and e-QTL genes contain a signal transduction system leading to the production of interferon-γ (IFN-γ), which is involved in immune and inflammatory reactions, and genes involved in B cell differentiation. These genes are assumed to play an important role in the development of PBC. In support of these data, an integrated pathway analysis of Japanese PBC susceptibility genes and analyses of PBC patient liver biopsy transcriptome data indicated that genes with characteristic expression patterns in PBC patients are primarily regulated by upstream factors such as *IFNG* and *CD40L* [64].

### 3.4. In Silico Drug Efficacy Screening

An in silico drug efficacy screening study using disease-susceptibility genes identified by GWAS has recently been reported for several diseases [79,80,81]. This method can identify the candidate molecular targets of drugs that show protein–protein interaction with the protein product of disease-susceptibility genes by using drug databases, the therapeutic target database, and the protein–protein interaction database. In addition to the drugs that are used for each disease, this method could potentially identify previously unexploited molecular targets of drugs. We identified immunotherapeutic drugs such as ustekinumab, abatacept, and denosumab; retinoids such as acitretin; and fibrates such as bezafibrate, as potential repurposed drug candidates for the treatment of PBC via in silico drug efficacy screening [39].

## 4. Conclusions

In this study, we summarized the PBC susceptibility genes reported as at December 2022 and discussed the latest post-GWAS approaches to identifying primary functional variants (causal variants) and effector genes. The understanding of the mechanisms by which these genetic factors mediate the pathogenesis of PBC has been expanded through the use of in silico gene set analyses, e-QTL and STRING database searching, and studies examining knock-in versions of cell lines constructed using genome-editing techniques such as CRISPR/Cas9.

Recent advances in informatics and genetic statistics methodologies have led to new analytical methods that utilize GWAS data. For example, SNP imputation analysis can be used to infer the genotypes of variants not loaded on a GWAS array by combining with thousands of individual whole-genome sequencing data. High-density association mapping, which involves case-control association studies for all of the variants in the disease-susceptible gene regions, can detect variants with *p*-values less than those of the GWAS lead variants located in the same LD blocks. *Protein kinase C β (PRKCB)* and chromosome 3q13.33 (including *ARHGAP31*, *TMEM39A*, *POGLUT1*, *TIMMDC1*, and *CD80*) were identified as the susceptibility gene loci for PBC in the Japanese population using high-density association mapping [41,42]. 

SNP imputation analysis can also be used for meta-analysis combining GWAS data that were analyzed by different GWAS arrays. In a recent genome-wide meta-analysis (meta-GWAS) of five European and two East Asian cohorts, SNP imputation analysis was performed [44,45]. By performing such SNP imputation analysis, it was possible to evaluate the association of primary functional variants with disease susceptibility, the correlation of primary functional variants with e-QTL, and the LD of primary functional variants with other variants. Thus, SNP imputation analysis is indispensable for post-GWAS analysis.

Whole-genome sequencing analysis of individual PBC patients can aid in the identification of disease-causing rare variants that have not been identified by GWASs and subsequent SNP imputation analyses. At present, this analysis has succeeded in detecting rare pathological variants in many Mendelian diseases and some immune-related diseases, such as cold medicine-related Stevens–Johnson syndrome [82]. Whole-genome sequencing analysis will be potentially useful in the identification of disease-causing or disease-modifying rare variants of PBC. 

Additionally, for more accurate post-GWAS analysis, protein–QTL analysis, an approach derived from a novel proteomics assay capable of measuring thousands of human protein analytes, enables accurate determination of correlations between variants and expression levels, without the uncertainty of e-QTL data that results from occasional non-correlations between mRNA and protein levels [83].

In the near future, re-analysis of existing GWAS data may yield important findings that have thus far been overlooked in the pathogenesis of multifactorial diseases such as PBC.

## Figures and Tables

**Table 1:** PBC susceptibility loci and e-QTL genes.

Chromosome	Location	Nearest Gene	GWAS-Lead SNP	*P* ^a^	e-QTL Genes (GTEx) ^b^	e-QTL Genes (ImmuNexUT) ^c^	Population	Ref
1	1p36.32	*MMEL1*	rs867436	2.99 × 10^−9^	-	-	European	[33]
	1p31.3	*IL12RB2*	rs6679356	4.85 × 10^−65^	*IL12RB2* (Whole Blood)	-	European	[32]
	1p13.1	*CD58*	rs10802191	3.68 × 10^−17^	-	*CD58* (T cell, B cell)	European, Asian	[43]
	1q23.1	*FCRL3*	rs945635	1.59 × 10^−8^	*FCRL1 (Whole blood, Spleen) * *FCRL3* (Whole blood, Spleen)	*FCRL1* (T cell, B cell, Monocyte) *FCRL2 *(T cell, B cell) *FCRL3* (T cell, B cell, NK cell, Neutrophil) *FCRL5* (B cell, DC)	European	[44]
	1p31.3	*DENND1B*	rs12123169	9.75 × 10^−18^	-	-	European	[35]
	1q32.1	*CACNA1S*	rs55734382	2.06 × 10^−9^	*GPR25* (Whole blood)	-	European	[44]
2	2p25.1	*LINC00299*	rs13416555	2.95 × 10^−8^	*LINC00299* (Liver)	*LINC00299* (NK cell) *ID2* (Monocyte)	International Meta	[44]
	2p23.3	*DNMT3A*	rs34655300	5.23 × 10^−10^	-	-	European	[44]
	2q21.3	*TMEM163*	rs859767	8.94 × 10^−16^	*TMEM163* (Whole Blood)	*TMEM163* (NK cell)	European, Asian	[44]
	2q32.2	*STAT4*	rs11889341	3.41 × 10^−31^	-	-	European, Asian	[35]
			RHM ^d^	-	-	-	Asian	[52]
	2q33.2	*CD28*	rs10581773	3.82 × 10^−13^	-	*CD28* (T cell)	Asian	[43]
3	3p24.3	*PLCL2*	rs1156336	7.13 × 10^−12^	*PLCL2* (Whole Blood)	*PLCL2* (T cell, Neutrophil)	European	[35]
	3p24.2	*RARB*	rs6550965	1.50 × 10^−14^	-	-	European	[44]
	3p22.1	*ULK4*	RHM ^d^	-	-	-	Asian	[52]
	3q13.33	*TIMMDC1*	rs2293370	2.05 × 10^−31^	*POGLUT1* (Whole Blood)	*POGLUT1* (T cell, Monocyte, DC)	European, Asian	[35]
	3q25.33	*IL12A*	rs589446	1.96 × 10^−55^	-	-	European	[32]
4	4q24	*NFKB1*	rs6533022	2.21 × 10^−32^	*MANBA * (Spleen, Liver)	*NFKB1 (T cell, Neutrophil, DC)* *MANBA* (T cell, NK cell, Monocyte, Neutrophil, DC) *UBE2D3* (T cell, Monocyte) *CISD2* (DC)	European, Asian	[35]
	4q24	*TET2*	rs2007403	6.19 × 10^−10^	-	-	European	[44]
5	5p13.2	*IL7R*	rs11742270	4.44 × 10^−24^	-	-	European, Asian	[35]
	5q21.1	*ST8SIA4*	rs60643069	2.48 × 10^−9^	-	-	International Meta	[44]
	5q31.3	*NDFIP1*	rs6874308	4.67 × 10^−8^	*NDFIP1* (Whole Blood, Liver)	*NDFIP1* (T cell, B cell, Monocyte, Neutrophil, DC) *GNPDA1* (T cell)	International Meta	[44]
	5q33.3	*IL12B*	rs2546890	4.61 × 10^−12^	-	*RNF145* (T cell, Monocyte)	European	[39]
6	6p21.32	*HLA genes*	rs7774434	2.72 × 10^−116^	*HLA-DRB6 (Whole Blood) * *HLA-DQA2 (Whole Blood) * *HLA-DRB1* (Whole Blood)	-	European, Asian	[44]
	6q21	*ATG5*	rs742108	3.16 × 10^−8^	-	*PRDM1* (T cell)	International Meta	[44]
	6q23.3	*LINC03004*	rs2327832	2.31 × 10^−10^	-	-	European	[39]
	6q27	*CCR6*	rs4709148	2.18 × 10^−10^	*CCR6 (Whole Blood)* *FGFR1OP (Whole Blood) * *RNASET2* (Whole Blood)	*CCR6* (T cell, B cell, DC) *FGFR1OP* (T cell, DC) *RNASET2* (T cell, B cell, NK cell, Monocyte, DC)	Asian	[44]
7	7p21.1	*ITGB8*	rs7805218	4.12 × 10^−8^	-	-	European	[44]
	7p14.1	*ELMO1*	rs60600003	4.70 × 10^−13^	-	-	European	[35]
	7q32.1	*TNPO3*	rs12531711	8.57 × 10^−41^	*TNPO5 (Whole Blood)* *IRF5* (Whole Blood)	-	European	[33]
	7q34	*ZC3HAV1*	rs370193557	9.37 × 10^−10^	-	-	European	[44]
8	8q24.21	*MYC*	rs4733851	4.98 × 10^−8^	-	-	International Meta	[44]
9	9q22.33	*ANP32B*	rs112500293	7.63 × 10^−9^	*ANP32B* (Whole Blood)	*TRIM14 * (T cell, DC)	European	[44]
	9q32	*TNFSF15*	rs1322057	5.48 × 10^−26^	-	*TNFSF15* (B cell, Monocyte, Neutrophil, DC) *TNFSF8 *(T cell, NK cell, Monocyte) *TNC* (T cell)	Asian	[40]
10	10q11.23	*WDFY4*	rs7097397	2.42 × 10^−10^	-	*WDFY4* (DC)	European	[44]
11	11p15.5	*DEAF1*	rs58523027	4.00 × 10^−8^	-	-	European	[44]
	11q13.1	*CCDC88B*	rs11601860	1.45 × 10^−10^	-	-	European	[35]
	11q23.1	*POU2AF1*	rs12419634	1.22 × 10^−13^	*COLCA1* (Spleen)	*POU2AF1 (B cell)* *COLCA1 (B cell) * *COLCA2 (B cell)*	European, Asian	[40]
	11q23.3	*CXCR5*	rs7119044	6.85 × 10^−38^	*TREH* (Liver) *DDX6* (Whole Blood)	*CXCR5* (B cell)	European, Asian	[35]
	11q24.3	*ETS1*	rs10893872	9.77 × 10^−9^	-	-	International Meta	[44]
12	12p13.31	*TNFRSF1A*	rs1800693	1.19 × 10^−16^	-	*TNFRSF1A* (T cell, NK cell, Neutrophil) *LTBR* (Neutrophil)	European	[35]
	12q24.12	*ATXN2*	rs35350651	5.50 × 10^−19^	-	-	European	[37]
13	13q14.11	*TNFSF11*	rs9533122	5.83 × 10^−13^	-	*TNFSF11* (NK cell)	European	[38]
	13q14.2	*DLEU1*	rs9591325	2.14 × 10^−19^	-	-	European	[39]
14	14q13.2	*SRP54*	rs799469	1.73 × 10^−9^	*SRP54 (Whole Blood)* *FAM177A1 * (Whole Blood)	*SPR54* (B cell, Neutrophil) PPP2R3C (Monocyte, Neutrophil, DC) *FAM177A1* (T cell, Monocyte, Neutrophil, DC) *KIAA0391* (T cell, NK cell, Monocyte, Neutrophil, DC) *BAZ1A* (Neutrophil) *PSMA6 *(Monocyte)	International Meta	[44]
	14q23.2	*KCNH5*	RHM ^d^	-	-	-	Asian	[52]
	14q24.1	*RAD51B*	rs3784099	8.31 × 10^−17^	-	-	European	[35]
	14q32.12	*RIN3*	rs72699866	2.89 × 10^−11^	-	-	European	[44]
	14q32.32	*EXOC3L4*	rs59643720	2.73 × 10^−38^	-	*TNFAIP2 * (B cell, Monocyte)	European	[35]
16	16p13.13	*CLEC16A*	rs12919083	3.63 × 10^−26^	-	-	European	[35]
	16p12.1	*IL21R*	rs1119132	4.09 × 10^−14^	-	*IL21R* (T cell, B cell, DC) *IL4R* (DC)	European	[43]
	16q22.1	*DPEP2*	rs79577483	1.23 × 10^−11^	*DPEP3 (Whole Blood) * *LCAT (Whole Blood) * *SLC12A4 (Whole Blood) * *DUS2 (Whole Blood) * *PRMT7 (Whole Blood) * *STRL (Whole Blood) * *ELMO3 (Whole Blood) * *FAM65A (Whole Blood) * *PARD6A* (Whole Blood) *PRMT7* (Spleen)	*DPEP2 (T cell, NK cell, Monocyte, DC)* *DUS2* (B cell, Monocyte, Neutrophil, DC) *SLC7A6* (T cell, Monocyte, Neutrophil, DC) *NFATC3* (T cell, B cell, NK cell, Neutrophil) *PLA2G15* (B cell, Monocyte) *DPEP3* (Monocyte, Neutrophil) *PRMT7* (T cell, B cell, NK cell, Monocyte) *DPRP2* (T cell, NK cell, Monocyte, DC) *SLC12A4* (Neutrophil, DC) *SMPD3* (B cell) *FBXL8* (B cell) *DDX28* (T cell) *PSKH1* (B cell)	European	[44]
	16q24.1	*IRF8*	rs11117432	2.82 × 10^−24^	-	-	European	[35]
17	17q12	*IKZF3*	rs71152606	8.36 × 10^−44^	-	*IKZF3* (T cell, NK cell) *ORMDL3* (T cell, B cell, DC) *GSDMB* (T cell, B cell, Monocyte, DC) *GSDMA* (T cell) *ZPBP2 *(B cel) *PGAP3* (B cell) *STARD3 *(T cell, Monocyte, DC)	European, Asian	[34]
	17q21.31	*HROB*	Chr17:44149348 A > C	8.16 × 10^−11^	-	-	European	[37]
18	18q22.2	*CD226*	rs1808094	1.66 × 10^−10^	*DOC6 * (Whole Blood, Spleen)	*CD226 (T cell, B cell, NK cell, DC)* *DOC6* (T cell, NK cell)	European	[44]
19	19p13.2	*TYK2*	rs2304256	4.43 × 10^−17^	*TYK2 (Whole Blood)* *AP1M2* (Whole Blood)	*TNK2* (T cell, B cell, NK cell, Monocyte, Neutrophil, DC) *PDE4A* (T cell, B cell, Monocyte) *ICAM3 *(Monocyte, Neutrophil) *CDC37* (T cell) *RAVER1* (Neutrophil)	European	[37]
	19q13.33	*SPIB*	rs3745516	2.65 × 10^−30^	*KAPSB * (Whole Blood)	*SPIB* (B cell, DC)	European	[34]
22	22q13.1	*SYNGR1*	rs137687	2.37 × 10^−23^	*SYNGR1* (Whole Blood, Liver, Spleen)	*SYNGR1* (T cell, B cell, NK cell, Monocyte, DC)	European	[35]
X	Xp11.23	*GRIPAP1*	rs7059064	6.20 × 10^−9^	*PCSK1N* (Whole Blood) *KCND1* (Whole Blood)	-	Asian	[45]

^a^ The Lowest *p*-value in the international meta-analysis (Mells et al, 2021), ^b^ e-QTL genes with GWAS-lead SNPs for liver and immunological organs (whole blood and spleen) registered in GTEX portal. ^c^ e-QTL genes with GWAS-lead SNPs registered in ImmuNexUT (*p* < 0.0001). ^d^ PBC susceptibility locus detected by the Regional Heritability Mapping (RHM) method. The genes shown in red are e-QTL genes that do not match the nearest gene.

## Data Availability

No new data were created or analyzed in this study. Data sharing is not applicable to this article.
